# AEG-1 silencing attenuates M2-polarization of glioma-associated microglia/macrophages and sensitizes glioma cells to temozolomide

**DOI:** 10.1038/s41598-021-96647-3

**Published:** 2021-08-30

**Authors:** Jing Li, Yuchen Sun, Xuanzi Sun, Xu Zhao, Yuan Ma, Yuzhu Wang, Xiaozhi Zhang

**Affiliations:** Department of Radiation Oncology, The First Affiliated Hospital of Xi’an Jiaotong University, Xi’an, 710061 China

**Keywords:** Computational biology and bioinformatics, CNS cancer, Tumour immunology, Cell signalling

## Abstract

Glioma is the most frequent primary malignancy in the brain; temozolomide (TMZ) is the first-line chemotherapeutic agent used to combat this tumor. We showed here that astrocyte elevated gene-1 (*AEG-1*) was overexpressed in glioma tissues and associated with a worse subtype and a poor prognosis. CCK-8 proliferation assays and clone formation experiments presented that *AEG-1* knockdown sensitizes glioma cells to TMZ. The *γH2AX* foci formation assays indicated that *AEG-1* silencing promotes TMZ-induced DNA damage in glioma cells. Glioma-associated microglia/macrophages (GAMs), the largest subpopulation infiltrating glioma, play important roles in the tumor microenvironment. Bioinformatics analyses and functional studies demonstrated that *AEG-1* silencing decreased M2-polarization of HMC3 microglia and the secretion of tumor supportive cytokines *IL-6* and *TGF-β1*. The expression of *AEG-1* was positively associated with M2 markers in glioma tissues varified by IHC staining. Based on the results of Affymetrix microarray and GSEA analyses, Western blot and Co-Immunoprecipitation assays were conducted to show that *AEG-1* activates Wnt/β-catenin signaling by directly interacting with *GSK-3β*. The co-localization of *AEG-1* and *GSK-3β* in the cytoplasm of glioma cells was detected through immunofluorescence staining. This study raises the possibility that targeting *AEG-1* might improve the efficiency of chemotherapy and reduce immunosuppressive M2 GAMs in glioma.

## Introduction

Glioma, the most frequent primary brain tumor, represents 81% of central nervous system (CNS) malignancies^1^. According to the histology and molecular characteristics, gliomas are categorized into grade I-IV by the World Health Organization (WHO)^2^. Despite accessorial advances in the therapeutic approach, the survival rate of grade IV gliomas (glioblastoma) patients remains only 5.8% at 5 years postdiagnosis^3^, with a median overall survival (OS) time of 15 months^4^. Current standard therapies are surgery, radiation, and chemotherapy. Temozolomide (TMZ) is the most prevalently used first-line drug for glioma patients, capable of crossing the blood–brain barrier and promoting apoptosis of glioma cells^5^. However, the development of chemoresistance impedes the efficacy of TMZ treatment. Thus, it is urgently needed to develop efficient strategies to increase the sensitivity to TMZ for glioma cells and improve patient prognosis.

Astrocyte elevated gene-1 (*AEG-1*), also known as Metadherin (*MTDH*), was originally identified induced in primary human fetal astrocytes infected with HIV-1 or treated with *TNF-α*^6^. Previous studies showed that *AEG-1* was involved in proliferation, metastasis, invasion, and chemoradiotherapy resistance in multiple types of cancer^7,8^. Moreover, *AEG-1* is the critical convergence point of diverse signaling pathways, such as PI3K/AKT, NF-κB, MAPK, and Wnt/β-catenin pathways^9^. It was reported that overexpressed *AEG-1* could decrease mRNA levels of *IL-6*, *IL-1β*, and *TNF-α* to suppress apoptosis in Kupffer cells^10^. Recently, *AEG-1* was revealed to be required in both tumor cells and tumor microenvironment (TME) macrophage activation to stimulate hepatocarcinogenesis^11^. However, the effects of *AEG-1* expression on glioma cells sensitivity to TMZ and immune infiltration remain ambiguous.

One of the most important features of glioma is the ability to evade the immune cells killing by creating an immunosuppression microenvironment, in which glioma-associated microglia/macrophages (GAMs) can be involved^12–14^. GAMs were identified as the largest subpopulation infiltrating human gliomas, occupying 30–50% of the tumor mass^14,15^. In addition, GAMs possess two different dynamic conditions of activation: the M1 pro-inflammatory phenotype, characterized by antitumor responses, and the M2 immunosuppressive phenotype, involved in tumor supportive responses conversely^16^. The two phenotypes are believed to reflect a spectrum of plastic functional states, instead of a set of discrete activation conditions^17^. It was revealed that GAMs could release numerous cytokines and signaling molecules to promote the proliferation and migration of glioma cells^13,18^. Glioma could recruit GAMs and regulate their M1/M2 polarization in turn^19,20^. Therefore, the further unraveling of the intracellular molecular interactions between glioma and GAMs will provide a crucial alternative treatment for glioma patients.

In this context, we reported herein the function of *AEG-1* in modulating the polarization of GAMs and improving sensitivity to TMZ for human glioma cells. It was illustrated that *AEG-1* activates Wnt/β-catenin signaling via targeting *GSK-3β*. In addition, we presented evidence that *AEG-1* silencing augments TMZ-induced DNA damage in glioma cells, providing a potential target for glioma therapy.

## Results

### Increased *AEG-1* expression level in glioma is correlated with a worse subtype and a poor prognosis

**Figure 1:**
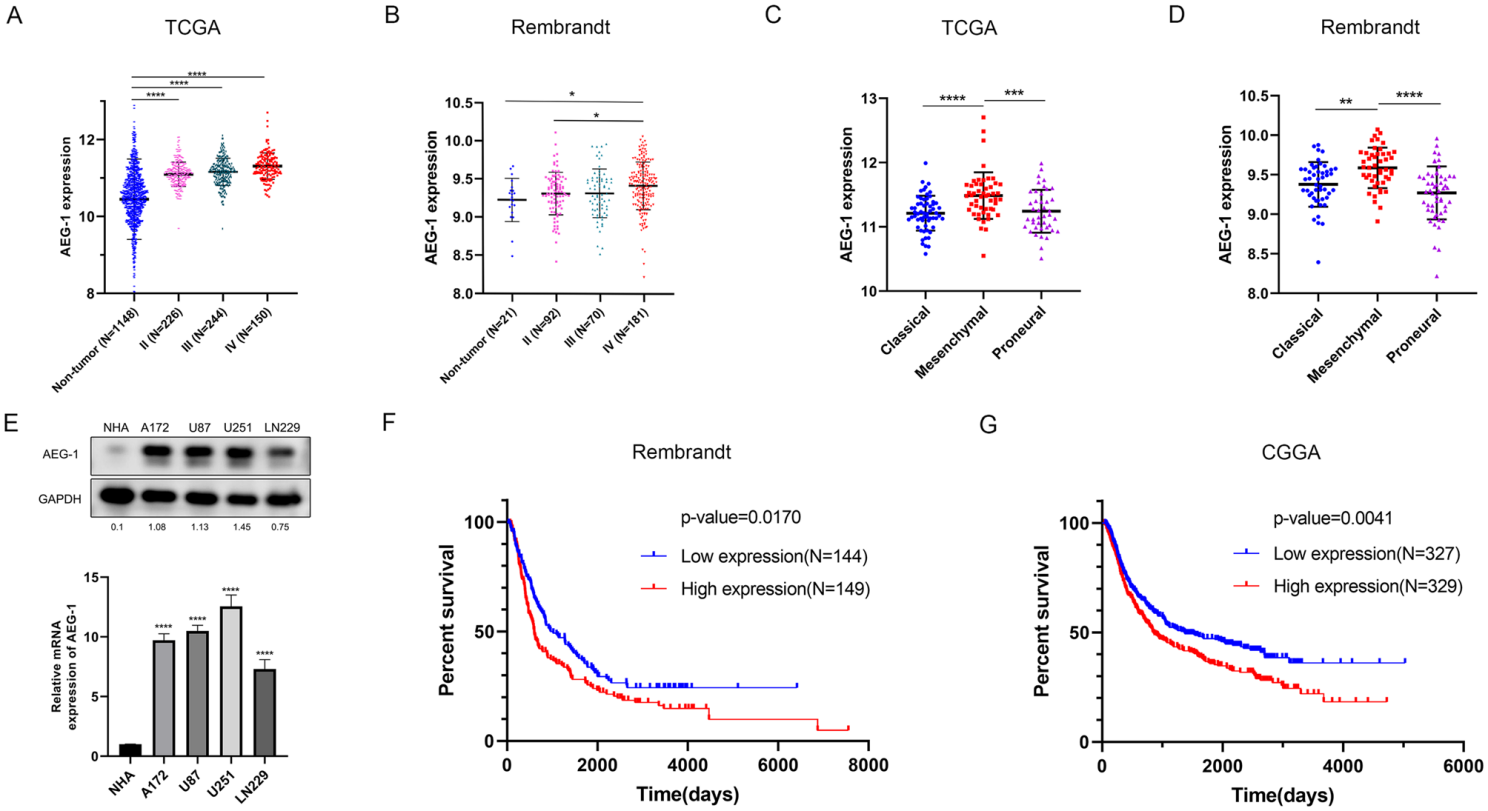
Expression of *AEG-1* in glioma and Kaplan–Meier plots. (**A**) *AEG-1* expression in different WHO grades of glioma patients from the TCGA database compared with non-tumor tissues from GTEx. (**B**) Expression levels of *AEG-1* in glioma patients compared with non-tumor tissues from the Rembrandt database. (**C**, **D**) *AEG-1* expression levels in three subtypes of glioma from TCGA and Rembrandt database. (**E**) mRNA and protein levels of *AEG-1* expression were examined by Real-time PCR and Western blot in NHA and glioma cell lines, respectively. *AEG-1* expression levels of all glioma cells were compared with that of NHA cells, calculated by ImageJ and GraphPad Prism 8.2.1 version. (**F**, **G)** Kaplan–Meier survival analyses of *AEG-1* expression and glioma patients’ overall survival in Rembrandt (n = 475) and CGGA database (n = 693). *P < 0.05, **P < 0.01, ***P < 0.001, ****P < 0.0001.

We found that the *AEG-1* expression in glioma tissues from the TCGA database was higher than non-tumor samples from Genotype-Tissue Expression (GTEx, Fig. 1A). For the Rembrandt database, although only glioma patients of WHO IV grade showed significantly more *AEG-1* expression than non-tumor tissues, a similar trend can be observed in lower-grade glioma (Fig. 1B). Based on gene expression profiles, glioma can be roughly divided into three subtypes: Proneural/Neural, Classical, and Mesenchymal^21^, in which the Mesenchymal subtype shows the worst prognosis^22^. Analyses in these subtypes showed that *AEG-1* was higher expressed in the Mesenchymal subtype (Fig. 1C, D). In addition, Western blot and Real-time PCR analyses were performed to measure the abundance of *AEG-1* expression in normal human astrocytes (NHA) and glioma cells. The results suggested that *AEG-1* expression was elevated in A172, U87, U251, and LN229 cells, relative to NHA (Fig. 1E). Kaplan–Meier survival curve of the Rembrandt indicated that patients with increased *AEG-1* expression had a shorter survival time (Fig. 1F). Furthermore, we identified a larger cohort of glioma patients in the CGGA database (N = 693). A similar result was observed that increased *AEG-1* expression was associated with a poor prognosis (Fig. 1G).

### Correlation between *AEG-1* expression and tumor-infiltrating immune cells in glioma

**Figure 2:**
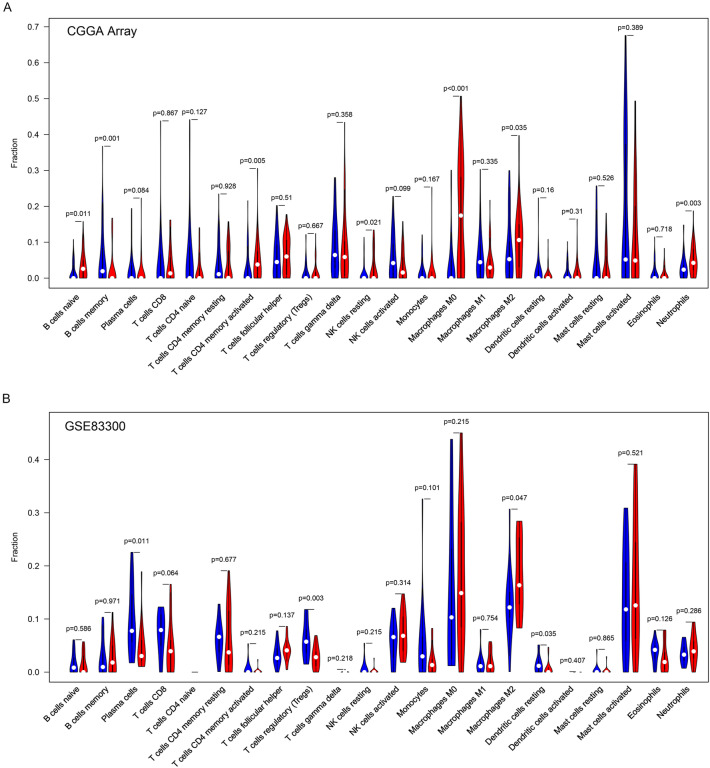
Correlation between *AEG-1* expression and tumor-infiltrating immune cells in glioma. Comparisons of immune cells infiltration between *AEG-1* high (red parts) and *AEG-1* low expression (blue parts) glioma tissues in CGGA array (**A**) and GSE83300 (**B**) cohort using CIBERSORT analyses [drawn by R 3.6.0 (https://cran.r-project.org/doc/FAQ/R-FAQ.html#Citing-R)].

To reveal the relationship between *AEG-1* expression and glioma immune evasion, CIBERSORT analyses were adopted to estimate the abundance of 22 types of immune cells in the CGGA array and GSE83300. We divided the samples into *AEG-1* high and low expression groups by the median cut-off value to maximally diminish the deviation. In the CGGA array, 7 types of immune cells (B cells naïve, B cells memory, T cell CD4 memory activated, NK cells resting, Macrophages M0, M2, and Neutrophils) were significantly different between *AEG-1* high and low expression groups (Fig. 2A, P < 0.05). Besides, the research in GSE83300 showed that *AEG-1* expression was correlated with the infiltration levels of Plasma cell (P = 0.011), Tregs (P = 0.003), Macrophage M2 (P = 0.047) and Dendritic cells resting (P = 0.035) (Fig. 2B). Collectively, *AEG-1* expression was associated with some types of tumor-infiltrating immune cells in glioma. Of further note, Macrophage M2 is the common significant result in the analyses for two different cohorts. The infiltration level of Macrophage M2 was higher in the *AEG-1* high expression group.

### Knockdown of *AEG-1* attenuates the generation of M2 glioma-associated microglia/macrophages (GAMs)

**Figure 3:**
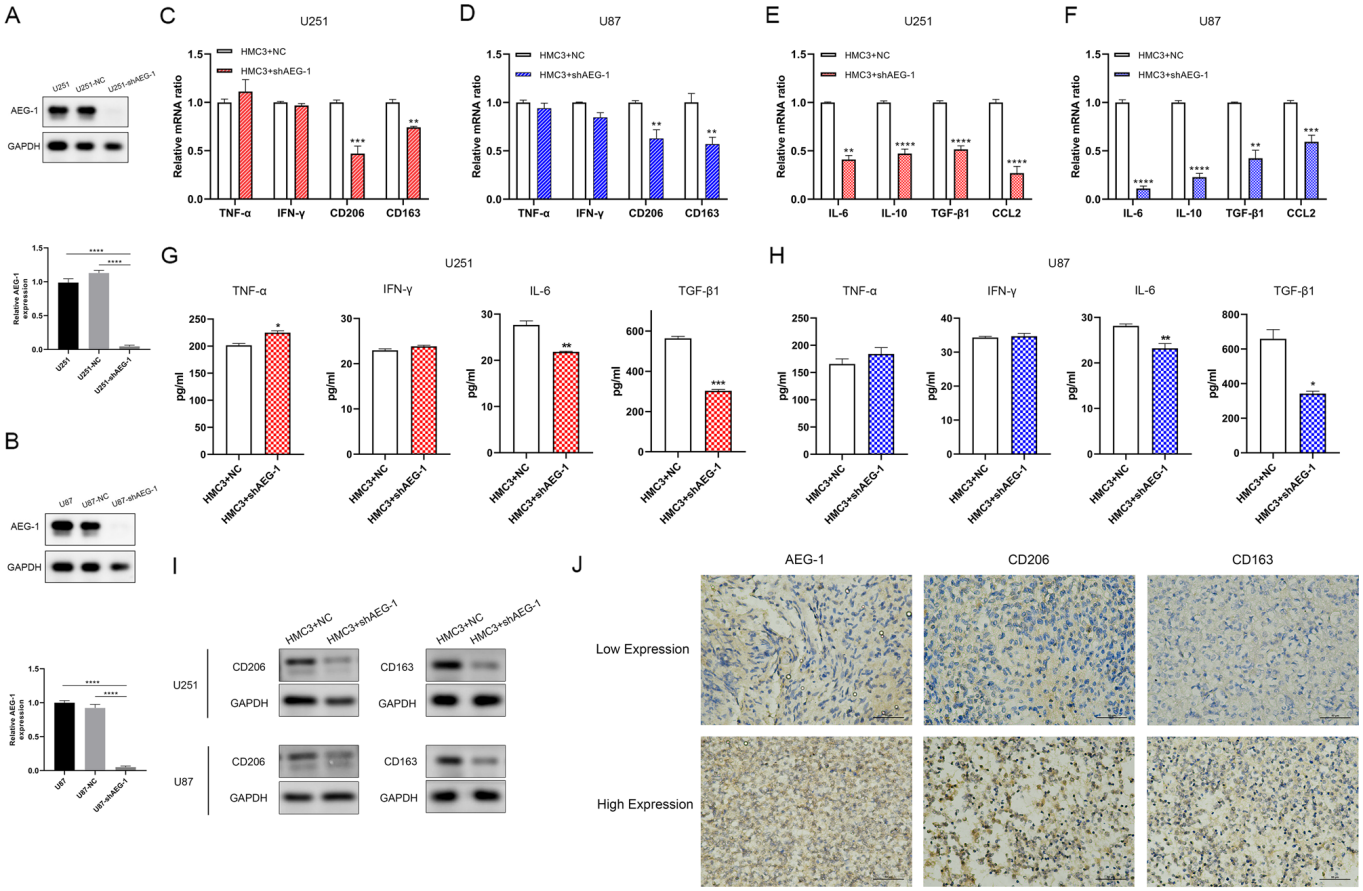
*AEG-1* silencing reduces the generation of M2 GAMs. (**A**, **B**) Validation of *AEG-1* knockdown in U251 and U87 cell lines following lentivirus transfection by Western blot. (**C**, **D**) The mRNA levels of M1 markers (*TNF-α, IFN-γ*) and M2 markers (*CD206*, *CD163*) in HMC3 microglia co-cultured with glioma NC and sh*AEG-1* cells were tested using Real-time PCR. (**E**, **F**) Real-time PCR analyses for mRNA levels of *IL-6, IL-10, TGF-β1,* and *CCL2* in HMC3 microglia co-cultured with NC and sh*AEG-1* glioma cells. (**G**, **H**) ELISA assays for the supernatant concentrations of M1 cytokines (*TNF-α, IFN-γ*) and M2 cytokines (*IL-6, TGF-β1*) in U251 and U87 NC and sh*AEG-1* cells co-cultured with HMC3 microglia. (**I**) Protein levels of M2 markers (*CD206*, *CD163*) in HMC3 microglia co-cultured with NC and shAEG-1 glioma cells were examined by Western blot. (**J**) Representative images of IHC staining for *AEG-1*, *CD206*, and *CD163* from human glioma patients. All data are the mean ± SD from three independent experiments. *P < 0.05, **P < 0.01, ***P < 0.001, ****P < 0.0001. *IHC* immunohistochemistry.

U251 and U87 cells were transfected with *AEG-1* shRNA lentivirus to generate *AEG-1* silencing cells, verified in protein levels (Fig. 3A, B). We postulated whether the expression of *AEG-1* in glioma cells regulated M2 GAMs, based on the bioinformatics analyses mentioned above. It was studied using HMC3 microglia and glioma cells co-culture system. We demonstrated that *AEG-1* knockdown in glioma cells substantially reduced the mRNA levels of M2 markers, *CD206* and *CD163* in co-cultured HMC3 microglia, to varying degrees in U251 and U87 co-culture systems. However, the mRNA levels of M1 markers, *TNF-α* and *IFN-γ* were not statistically significant between HMC3 cells co-cultured with NC and sh*AEG-1* glioma cells groups (Fig. 3C, D). In accordance, M2 cytokines mRNA levels of *IL-6, IL-10, TGF-β1,* and *CCL2* were significantly decreased in the *AEG-1* knockdown group (Fig. 3E, F). Next, we detected the secreted cytokines of HMC3 in the presence of NC and sh*AEG-1* glioma cells. Comparatively, M2 cytokines, *IL-6* and *TGF-β1* in the supernatant of HMC3 co-cultured with sh*AEG-1* U251 and U87 cells, were lower than control samples. HMC3 co-cultured U251 sh*AEG-1* cells showed slightly increased *TNF-α* cytokine level and similar *IFN-γ* level compared with the control group. For the U87 cells co-culture system, there was no significant difference in the concentration of M1 cytokines, *TNF-α* and *IFN-γ*. (Fig. 3G, H). A similar observation was made in protein levels of M2 markers. *CD206* and *CD163* protein levels were lower in the HMC3 microglia co-cultured sh*AEG-1* glioma cells (Fig. 3I). To further validate this phenomenon, we conducted immunohistochemistry staining of tissue samples from 50 patients with glioma. The results indicated that higher expression of *AEG-1* in glioma was positively correlated with the infiltration of *CD206*^+^ (r = 0.443, P = 0.001) and *CD163*^+^ (r = 0.333, P = 0.018) M2 GAMs (Fig. 3J, Table 1). Together, these data suggest that knockdown of *AEG-1* impaired the generation of M2 GAMs in glioma.

**Table 1:** Correlation between *AEG-1* expression and *CD163*/*CD206*^+^ macrophage.

	*AEG-1* high expression	*AEG-1* low expression	χ^2^	r	P
***CD163***					
High	20	5	5.556	0.333	0.018
Low	12	13			
***CD206***					
High	25	6	9.810	0.443	0.001
Low	7	12			

### *AEG-1* silencing improves the sensitivity of glioma cells to temozolomide

**Figure 4:**
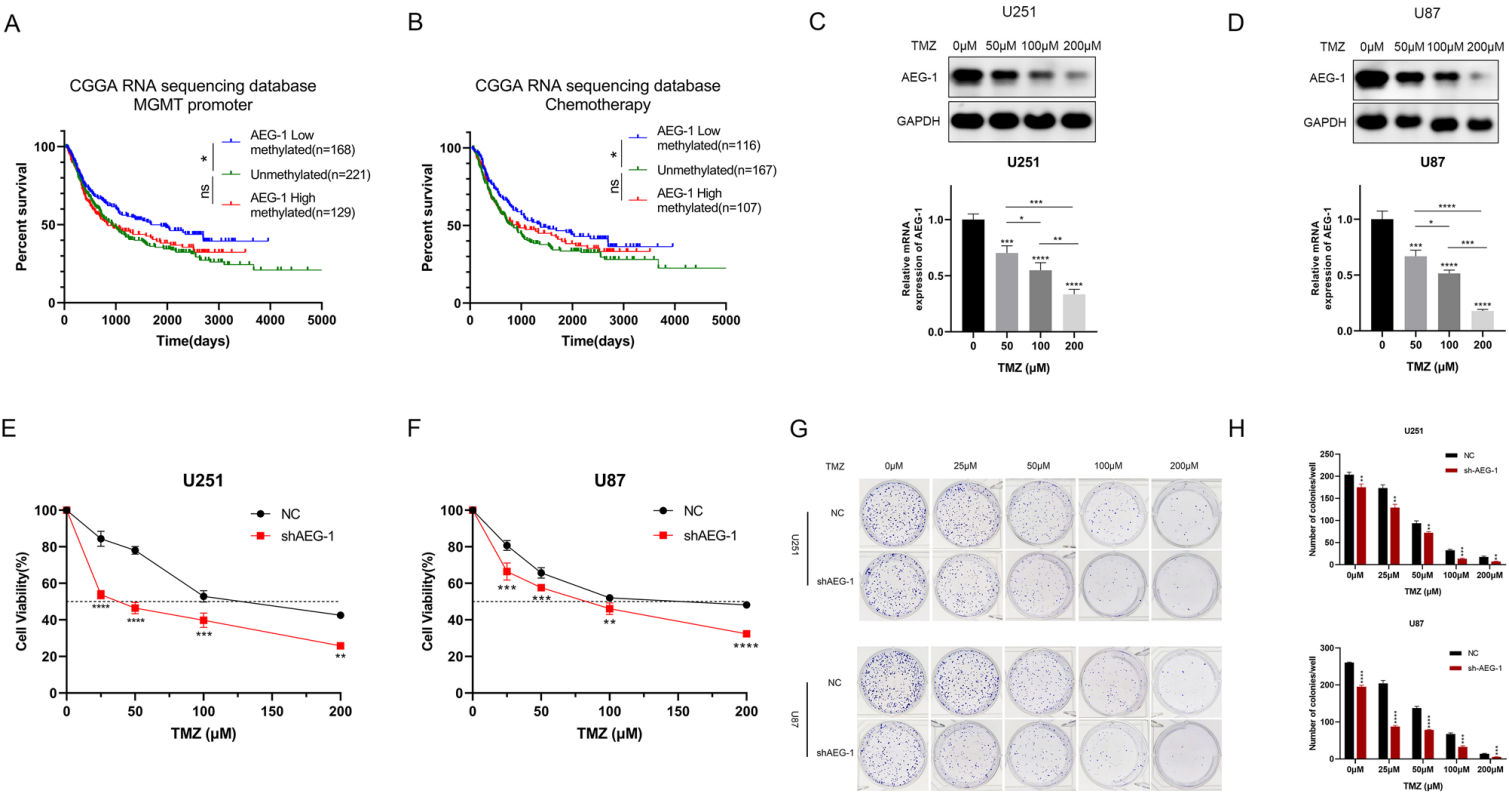
Knockdown of *AEG-1* sensitizes glioma cells to TMZ. (**A**) Kaplan–Meier survival analyses of three cohorts: low *AEG-1* expression with *MGMT* promoter methylated, *MGMT* promoter unmethylated, and high *AEG-1* expression with *MGMT* promoter methylated glioma patients in CGGA database. (**B**) The same analyses were made in patients from the CGGA database who accepted chemotherapy. (**C**, **D**) The mRNA and protein levels of *AEG-1* in U251 and U87 cells treated with TMZ were detected by Real-time PCR and Western blot, respectively. (**E**, **F**) CCK-8 assays of glioma cell lines following NC or sh*AEG-1* treated with TMZ at 72 h incubation. (**G**, **H**) Colony formation experiments of glioma U251 and U87 cells following NC or sh*AEG-1* treated with TMZ at different concentrations. The results were calculated using ImageJ and GraphPad Prism 8.2.1 software. All data are the mean ± SD from three independent experiments. *P < 0.05, **P < 0.01, ***P < 0.001, ****P < 0.0001. *TMZ,* temozolomide.

A common cognition has been reached that *MGMT* promoter methylation usually implied a better prognosis and increased TMZ sensitivity in glioma patients^23^. To investigate whether *AEG-1* expression can affect the chemosensitivity of glioma cells to TMZ, survival curves of the CGGA sequencing database were performed as the preliminary study. For all glioma patients or those who received chemotherapy, *MGMT* promoter methylated patients with *AEG-1* low expression had a survival advantage compared with unmethylated ones. However, there was no significant difference between the survival time of *MGMT* promoter methylated patients with *AEG-1* high expression and unmethylated ones (Fig. 4A, B). The analyses indicated that patients with high *AEG-1* expression cannot benefit from TMZ chemotherapy, even with *MGMT* promoter methylation.

Next, we examined the mRNA and protein levels of *AEG-1* in glioma cells treated with TMZ. Following TMZ treatment at increasing concentrations, *AEG-1* expression was notably decreased (Fig. 4C, D). For U251 cells, the IC50 value was mitigated from 138 to 37 μM post *AEG-1* knockdown (Fig. 4E). A similar observation was made in U87 cells that silencing *AEG-1* declined IC50 from 145 to 74 μM compared with control (Fig. 4F). The results of the CCK-8 proliferation assay were also supported by those of the clone formation assay (Fig. 4G, H).

### *AEG-1* silencing enhances TMZ-induced DNA damage in glioma cells

**Figure 5:**
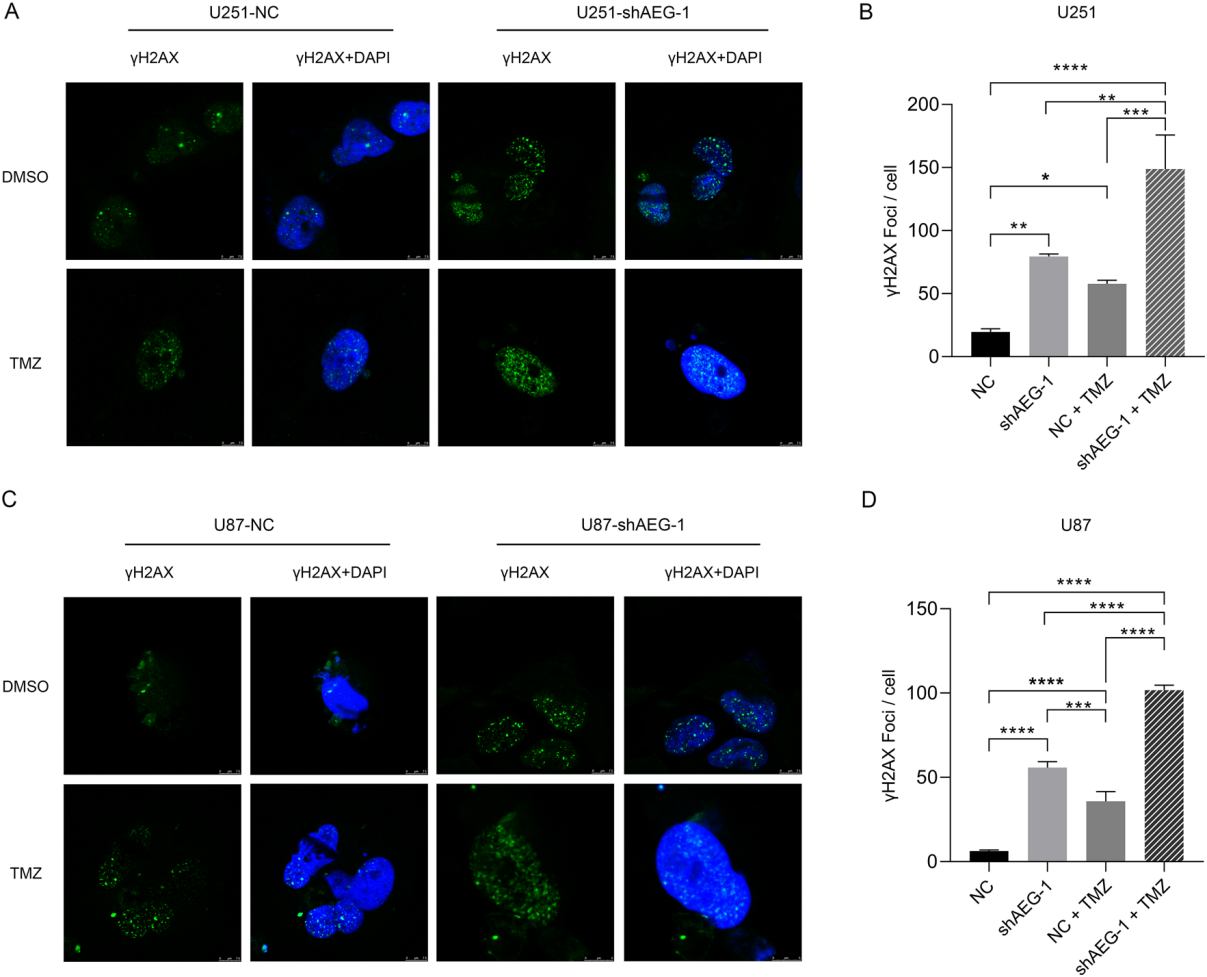
*AEG-1* silencing enhances TMZ-induced DNA damage in glioma cells. (**A**, **C**) U251-NC, U251-sh*AEG-1*, U87-NC, and U87-sh*AEG-1* cells were given with either DMSO or TMZ (50 μM) treatment for 48 h. Immunofluorescence staining of *γH2AX* foci (green) and DAPI (blue) was performed. The numbers of *γH2AX* foci per cell were calculated and showed as histograms (**B**, **D**). Three fields were quantified using ImageJ for each sample. Data were presented as mean ± SD from three independent experiments. *P < 0.05, **P < 0.01, ***P < 0.001, ****P < 0.0001. *TMZ,* temozolomide.

To investigate the effect of *AEG-1* expression on the DNA damage status of glioma cells after TMZ treatment, the number of *γH2AX* foci was examined by immunofluorescence staining. The results showed increased levels of *γH2AX* foci in the sh*AEG-1* group than NC group. Moreover, TMZ treatment (48 h) in glioma cells led to more *γH2AX* foci than DMSO control cells. This effect was significantly augmented in the *AEG-1* silencing group of U251 (Fig. 5A, B) and U87 cells (Fig. 5C, D). Therefore, the results revealed that down-regulation of *AEG-1* enhances the DNA damage induced by TMZ in glioma cells.

### *AEG-1* activates Wnt/β-catenin signaling in glioma cells via targeting *GSK-3β*

**Figure 6:**
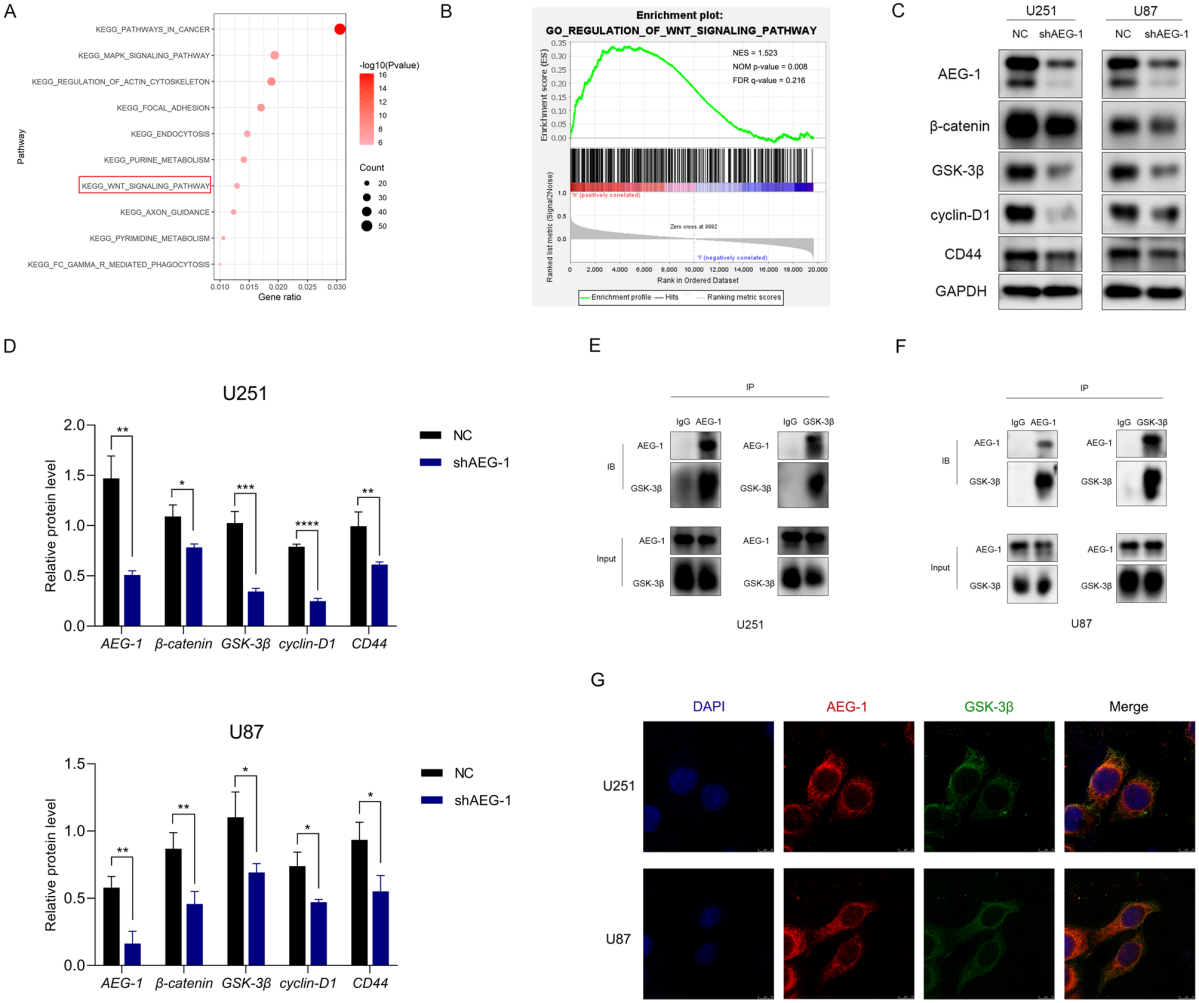
*AEG-1* activates Wnt/β-catenin signaling via targeting *GSK-3β* in glioma cells. (**A**) Enriched top 10 KEGG pathways for Affymetrix microarray of *AEG-1* NC (N = 3) and KD (N = 3) glioma cells. 22 DEGs were enriched in KEGG_WNT_SIGNALING PATHWAY. P-value = 1.02E−06. [drawn by R 3.6.0 (https://cran.r-project.org/doc/FAQ/R-FAQ.html#Citing-R)]. (**B**) Enrichment plot of the Wnt signaling pathway from GSEA; ‘h’ and ‘l’ represented *AEG-1* high and low expression, respectively. NES = 1.523, NOM P-value = 0.008, FDR q-value = 0.216 [drawn by GSEA tool (version 4.1.0)]. (**C**) Western blot bands of *AEG-1*, *β-catenin*, *GSK-3β*, *cyclin D1*, and *CD44* in NC and sh*AEG-1* glioma cell lines. (**D**) Relative protein abundance was calculated by ImageJ and GraphPad Prism 8.2.1 software. (**E**, **F**) In U251 and U87 cells, Co-IP assays showed the direct interaction of *AEG-1* and *GSK-3β*. (**G**) Immunofluorescence assays were used to detect the localization of *AEG-1* (red) and *GSK-3β* (green) in U251 and U87 cells. All data were presented as the mean ± SD from three independent experiments. *P < 0.05, **P < 0.01, ***P < 0.001, ****P < 0.0001.

*AEG-1* has been reported to interact with *β-catenin* in colorectal carcinoma^24^ and glioma stem cells^25^. To further identify the mechanism of *AEG-1* function, we conducted an Affymetrix microarray of negative control (NC) and *AEG-1* knockdown (KD) glioma cells. Based on the gene information of KEGG pathways, the top 10 enriched pathways for differentially expressed genes (DEGs) between NC and KD groups were listed, including KEGG_WNT_SIGNALING_PATHWAY (Fig. 6A, Supplementary Table S1). Also, GSEA analysis showed that *AEG-1* expression may be related to the regulation of the Wnt signaling pathway (Fig. 6B). The expression levels of Wnt-related genes, including *β-catenin*, *GSK-3β*, *cyclin D1*, and *CD44* were all decreased in *AEG-1* knockdown glioma cells compared to that in the control cells (Fig. 6C, D). Surprisingly, Co-IP experiments in our study indicated that *AEG-1* was unable to bind with *β-catenin* directly (Supplementary Fig. S1). Nevertheless, in U251 and U87 cells, IP of *AEG-1* pulled down endogenous *GSK-3β* and vice versa (Fig. 6E, F). The accumulated co-localization of *AEG-1* and *GSK-3β* mainly in the cytoplasm of glioma cells was detected by immunofluorescence staining (Fig. 6G). Thus, *AEG-1* targets *GSK-3β* and activates Wnt/β-catenin signaling in glioma cells.

## Discussion

Current standard treatment regimens of glioma include maximum surgical resection and adjuvant chemoradiotherapy. However, it is difficult to achieve complete surgical resection to avoid the additional injury to the normal brain tissues in clinical practice^1^. Most glioma patients develop acquired chemoresistance, leading to treatment failure. Thus, strategies are urgently required to enhance glioma cells sensitivity to chemotherapeutic agents, specifically for TMZ. Previously, we reported that *AEG-1* overexpression in glioma was correlated with advanced clinical stage and higher WHO grade^26,27^. *AEG-1* was associated with numerous human malignancies, such as non-small-cell lung cancer^28^, ovarian cancer^29^, and hepatocellular carcinoma^30^. Proliferation, migration, invasion, stemness, and chemoradiotherapy resistance were included in *AEG-1* evoked malignant hallmarks in tumor cells^26,28–31^. Here, we identified *AEG-1* was higher expressed in glioma than non-tumor tissues and related to a worse subtype and a poor prognosis in glioma patients from different cohorts.

TMZ is the standard first-line treatment for glioma patients, acting as an alkylating drug that methylates O^6^ and N^7^ position of guanine, O^3^ position of adenine^32^. *MGMT* could directly reverse O^6^-meG, the prime contribution of cytotoxicity, which can be induced by *MGMT* methylation^33^. Therefore, it was widely believed that TMZ resistance in glioma patients was associated with *MGMT* methylation condition. Besides, factors such as P4HB^34^, ALDH1A1^35^, and poly (ADP-ribose) polymerase^36^ have been reported to be related to TMZ resistance in glioma cells. TMZ treatment causes single and double-strand DNA breaks, inducing G2/M cell cycle arrest and apoptosis of cells^37^. In our previous report, *AEG-1* facilitates homologous recombination of DNA damage induced by radiation in glioma cells, indicating the vital role of *AEG-1* in DNA damage response^27^. In this work, survival analyses of glioma patients, CCK-8 assays, and colony formation experiments showed that *AEG-1* knockdown significantly sensitizes glioma cells to TMZ. However, *AEG-1* silencing does not affect *MGMT* expression (Supplementary Fig. S2). We assumed a mechanism by which AEG-1 silencing shifts the *MGMT* methylation condition. Further investigations are ongoing. By testing the formation of *γH2AX* foci, our results showed that *AEG-1* silencing increases TMZ-induced DNA damage in glioma cells.

Emerging studies have presented the TME of glioma plays a critical role in tumorigenesis and progression^38^. The interaction between tumor cells and TME may contribute to tumor immune evasion and further worse development^39,40^. GAMs were recognized as one of the abundant cell types in the glioma microenvironment and had been shown to promote tumor progression. Notably, the HMC3 microglia and glioma cells involved co-culture system has been gradually applied in immuno-oncology research^17,41^. In addition, we believe that GAMs are highly heterogeneous and there are not only two simple types of GAMs in the glioma niche. Here, we employed “M1” and “M2” polarization to roughly represent tumor-suppressive and tumor-supportive GAMs, respectively, that may contain a spectrum of subpopulations. This study showed that knockdown of *AEG-1* impaired M2-polarization of GAMs.

To reveal the underlying molecular mechanisms of *AEG-1* in glioma, microarray and GSEA analyses indicated that *AEG-1* expression was correlated with the Regulation of Wnt signaling pathway. This study showed that *AEG-1* silencing inhibits Wnt/β-catenin signaling by targeting *GSK-3β* in glioma cells. Wnt/β-catenin signaling has been validated to regulate tumor cell proliferation, migration, invasion, tissue homeostasis, stemness maintenance, and therapeutic resistance^42–44^. Tao found that Wnt-induced protein-1 is crucial for maintaining tumor-supportive M2 TAMs in GBM^39^. Activation of Wnt/β-catenin signaling in melanoma was identified to inhibit T cell infiltration, leading to tumor growth and immunotherapy resistance via reducing CCL4 secretion^45^. Also, activation of β-catenin signaling decreased the infiltration of T cells to the tumor, whereas the chemokine *CXCL10* had the opposite effect^46^. Deng identified β-catenin as a transcription factor of *PD-L1* expression^47^. Consistent with the reduction of *β-catenin* abundance in *AEG-1* silencing glioma cells, a large restraint of *PD-L1* expression (Supplementary Fig. S3) and an increased *CXCL10* expression (Supplementary Fig. S4) were observed, implying that *AEG-1* may play a potentially broader role in tumor immune evasion which worth further investigation.

Nevertheless, there are certain limitations in the current study. Whether suppression of Wnt/β-catenin signaling contributes to the promotion of glioma cells sensitivity to TMZ and reduction of M2 GAMs still needs further research. Interactions between *AEG-1* expression and *MGMT* methylation should be explored. It is encouraging that *AEG-1* silencing may affect cytotoxic immune cells infiltration due to resulting in a diminished *PD-L1* and an increased *CXCL10* expression. These issues worth in-depth study.

## Materials and methods

### Data collection

The gene expression and subtypes data for glioma were obtained from the TCGA database (https://tcga-data.nci.nih.gov/tcga/). Data of normal brain tissue samples were obtained from Genotype-Tissue Expression (GTEx, https://gtexportal.org/home/). The mRNA Sequencing, mRNA microarray, and corresponding clinical data were downloaded from the Chinese Glioma Genome Atlas (CGGA, http://www.cgga.org.cn/). Expression profiles and clinical information of Repository for Molecular Brain Neoplasia Data (Rembrandt) were also downloaded from CGGA (added on June 14, 2020). Dataset GSE83300, with 50 glioma tissue samples, was acquired from Gene Expression Omnibus (GEO, https://www.ncbi.nlm.nih.gov/geo/). Patients with survival < 30 days were removed from the analyses.

### Assessment for immune infiltration in TME of glioma

Based on the gene expression profile, CIBERSORT (http://cibersort.stanford.edu/) was utilized to provide the estimation for the abundance of 22 types of infiltration immune cells in TME of glioma^48^. The median expression of *AEG-1* was set to be the cut-off value.

### Gene set enrichment analysis (GSEA)

GESA was performed to search the significant pathways for the differentially expressed genes (DEGs) between *AEG-1* high and low expression groups of glioma samples from the TCGA database. In GSEA 4.1.0, the number of permutations was set to be 1000. The gene set database c5.go.v7.2.symbols.gmt was from the website. The high or low *AEG-1* expression level was set as a phenotype label.

### Cell culture, lentiviral vector, and transfection

Human glioma cell lines (A172, U87, U251, and LN229) and human microglia cell line HMC3 (ATCC^®^CRL-3304) were purchased from the Cell Bank of the Chinese Academy of Science Typical Culture Preservation Committee (Shanghai, China). Normal human astrocytes NHA were kindly provided from the medical department of Xi’an Jiaotong University. Cells were maintained in Dulbecco’s Modified Eagle’s Medium (DMEM; HyClone) supplemented with 10% fetal bovine serum (FBS; Gibco) and 1% penicillin–streptomycin (HyClone) in a 37 °C humidified incubator with 5% CO_2_.

The *AEG-1* short hairpin (shRNA) lentivirus vector was synthesized by GeneChem Co., Ltd (Shanghai, China) to silence *AEG-1* expression. The target sequence of *AEG-1* shRNA was 5′-AACTTACAACCGCATCATT-3′. The negative control (NC) lentivirus sequence was 5′-TTCTCCGAACGTGTCACGT-3′. The transfection was carried out in accordance with the manufacturer’s instructions.

### Cell viability assay

Cell viability was detected by Cell Counting Kit-8 assays (Topscience). 2500 control and sh*AEG-1* glioma cells per well, treated with different concentrations of TMZ (0, 25, 50, 100, 200 μM), were seeded into 96-well plates. TMZ (Topscience) was dissolved using DMSO (Sigma). At the indicated time point of 72 h, CCK-8 regents (10 μl per well) were added and incubated at 37 °C for another 3 h. Then, the OD value at 450 nm was measured to determine TMZ inhibition rates in different groups of glioma cells. The group of 0 μM TMZ was set to be a control.

### Colony formation assay

The colony formation assay was carried out to ascertain the effects of *AEG-1* knockdown and/or TMZ treatment on glioma cells. Briefly, manipulated U251 and U87 cells were incubated in 6-well plates at a density of 1000 cells per well. 12 h later, different concentration of TMZ (0, 25, 50, 100, 200 μM) was supplemented for 3 days. The colonies were fixed with methanol and then stained with 1% crystal violet for 20 min after 14 days of incubation.

### Cell co-culture and HMC3 polarization

Lentivirus manipulated U251 and U87 cells were co-cultured with HMC3 microglia at a density of 1:5 in 6-well plates (Nunc™ Polycarbonate Cell Culture Inserts in Multi-Well Plates with 0.4 μm aperture). Glioma cells (10^5^ cells per well) were seeded on the upper layer of the co-culture chamber. Simultaneously, HMC3 cells (5 × 10^5^ cells per well) were incubated on the lower layer of the chamber. After 72 h, the total RNA of HMC3 microglia was collected to analyze their M1, M2 phenotypes^41^ using Real-time quantitative PCR technique. The protein of HMC3 microglia was extracted to test the M2 phenotype by Western blot analysis. Furthermore, the supernatant was also gathered to determine the M1, M2 cytokines secreted into the culture medium via ELISA assay.

### Real-time quantitative PCR

Total RNA from cells was extracted using the Fastagen200 kit (Fastagen). NanoDrop 3000 was applied to qualify the RNA concentration. cDNA was synthesized from 1.0 μg total RNA in a 20 μl reaction system using Evo M-MLV RT Kit with gDNA Clean for qPCR (Accurate Biotechnology). RT-PCR was performed using 2* RealStar Green Fast Mixture (GeneStar Technology) as follows: denaturation at 95 °C for 2 min and 40 cycles of 95 °C for 15 s, 60 °C for 30 s, and 72 °C for 30 s. Mentioned primer sequences were listed in Table 2. GAPDH was applied as the reference gene.

**Table 2:** Primer sequences employed in this study.

Gene	Forward	Reverse
*AEG-1*	AAATAGCCAGCCTATCAAGACTC	TTCAGACTTGGTCTGAAGGAG
*GAPDH*	GAAGAGAGAGACCCTCACGCTG	ACTGTGAGGAGGGGAGATTCAGT
*TNF-α*	CCGAGGCAGTCAGATCATCTT	AGCTGC CCC TCA GCT TGA
*IFN-γ*	TGTAGCGGATAATGGAACTCTTTT	AATTTGGCTCTGCATTATT
*CD206*	GCAGAAGGAGTAACCCACCC	TGGCAAATGAAGGCGTTTGG
*CD163*	TCCACACGTCCAGAACAGTC	CCTTGGAAACAGAGACAGGC
*IL-6*	ACTCACCTCTTCAGAACGAATTG	CCATCTTTGGAAGGTTCAGGTTG
*IL-10*	CGAGATGCCTTCAGCAGAG	CGCCTTGATGTCTGGGTCTT
*TGF-β1*	CAAGGGCTACCATGCCAACT	AGGGCCAGGACCTTGCTG
*CCL2*	AGGTGTCCCAAAGAAGCTGT	ACAGAAGTGCTTGAGGTGGT

### Enzyme-linked immunosorbent assay (ELISA)

All ELISA kits were acquired from Multi Science (LIANKE) biotech. The concentration of M1 markers in the collected supernatant was detected by human *TNF-α* ELISA Kit (70-EK182) and Human *IFN-gamma* ELISA Kit (70-EK180), respectively. Human *IL-6* ELISA Kit (70-EK106/2) and Human/Mouse/Rat *TGF-β1* ELISA Kit (70- EK981) were used to quantify the M2 markers of the supernatant in the co-culture system. The experiments were performed in triplicates and according to the manufacturer's protocol.

### Immunohistochemistry (IHC)

The experiments, including any relevant details, were approved by the Ethics Committee of the First Affiliated Hospital of Xi’an Jiaotong University. The study was performed in accordance with relevant guidelines and regulations. 50 patients with glioma were enrolled in the research. All patients signed a written informed consent before participating in the study. IHC staining was performed as previously described^27^. Slides were incubated with the primary rabbit antibodies, including anti-*AEG-1* (13860-1-AP, Proteintech, 1:400), anti-*CD206* (GB13438, Servicebio, 1:800), and anti-*CD163* (GB11340-1, Servicebio, 1:1000).

### Microarray experiment

Total RNA from negative control (NC) and *AEG-1* knockdown (KD) glioma cells was extracted using TRIzol reagent (Invitrogen) and tested by NanoDrop 2000 and Agilent Bioanalyzer 2100. cDNA was synthesized with a High-Capacity cDNA reverse transcription kit (ThermoFisher), which was then hybridized by GeneChip Prime View Human Gene Expression Array (Affymetrix). The GeneChip was washed and stained by GeneChip Fluidics Station 450. Finally, GeneChip Scanner 3000 was used to scan the microarray chip.

### Western blot analysis

Total protein from glioma cells was extracted using RIPA lysis buffer (Sigma Aldrich) mixed with protease inhibitor. Subsequently, the protein was quantified with BCA Protein Assay Kit (Sigma Aldrich). Proteins were separated through standard 10% SDS-PAGE and then transferred adequately onto PVDF membranes (Millipore), incubated with primary antibodies (Proteintech) at 4 °C overnight. The membranes were washed with TBST solution, followed by incubated with secondary antibodies for 1 h at room temperature the next day. The protein bands were visualized using an ECL kit (Millipore).

### Co-immunoprecipitation (Co-IP)

U251 and U87 cells were lysed at 4 °C for 20 min using RIPA buffer, supplemented with protease inhibitor. After 12,000 rpm centrifugation, the supernatant was precleared with protein A/G beads (Santa Cruz Biotechnology) on ice for 30 min to reduce non-specific binding. *AEG-1* and *GSK-3β* rabbit antibody (Proteintech) were then added overnight under 4 °C shaking, respectively. Rabbit IgG (Cell Signaling Technology) acted as a negative control. The complex of antibody and antigen was dragged out of the lysates using protein A/G agarose beads. The beads were washed by novel RIPA lysis buffer three times the following day. Finally, resuspend the beads using a 2 × loading buffer. The Western blot procedure has been described previously.

### Immunofluorescence staining

Cells were seeded on glass-bottom cell culture dishes with a 20 mm-diameter (Corning) and grown to 30% confluence. Cells were fixed with 4% paraformaldehyde for 15 min, followed by washing with PBS three times. Then, cells were permeated by 0.5% Triton X-100 and blocked by 5% BSA for both 30 min at room temperature. Cells were incubated with rabbit anti-*AEG-1* antibody (Proteintech, 1:1000), mouse anti-*GSK-3β* antibody (Cell Signaling Technology, 1:1000), and rabbit anti-*γH2AX* (Cell Signaling Technology, 1:400), diluted using 1% FBS, at 4 °C overnight. Goat anti-rabbit Cy3-conjugated, FITC-conjugated secondary antibody, and goat anti-mouse Alexa Fluor Plus 488-conjugated secondary antibody (Invitrogen) were added and incubated for 1 h at 37 °C away from light. And then, Antifade Mounting Medium with DAPI (Beyotime Biotechnology) was supplemented for nuclear staining. The samples were imaged using Leica TCS SP5 confocal microscope.

### Statistical analysis

All statistical analysis and data visualization were performed using GraphPad Prism (version 8.2.1), ImageJ, SPSS 22.0, and R 3.6.0. The two-tailed Student’s *t* test was used to test the differences between two groups. One-way ANOVA was conducted to compare the differences among multiple groups. Correlation analyses among *AEG-1* and M2 markers were determined by Spearman rank-order correlation. Survival curves were performed using the Kaplan–Meier analysis. P < 0.05 was regarded as statistically significant.

## Supplementary Information

Supplementary Information.

## Data Availability

All data generated and analyzed during this study are included in this article and Supplementary Information files are available from the corresponding author on reasonable request.
