# Supplementary material for: AEG-1 silencing attenuates M2-polarization of glioma-associated microglia/macrophages and sensitizes glioma cells to temozolomide
Source: Sci Rep. 2021 Aug 30;11:17348. doi: 10.1038/s41598-021-96647-3 (PMC8405821; doi:10.1038/s41598-021-96647-3)

AEG-1 silencing attenuates M2-polarization of glioma-associated microglia/macrophages and sensitizes glioma cells to temozolomide

Jing Li[1], Yuchen Sun[1], Xuanzi Sun[1], Xu Zhao[1], Yuan Ma[1], Yuzhu Wang[1], and Xiaozhi Zhang[1*]

1. The First Affiliated Hospital of Xi'an Jiaotong University, Department of Radiation Oncology, Xi'an, 710061, China.

***Correspondence author:** Xiaozhi Zhang, E-mail: Zhangxiaozhi@xjtu.edu.cn.

Supplementary Fig. S1

Co-IP assays of *AEG-1* and *β-catenin* in U251 and U87 cells.

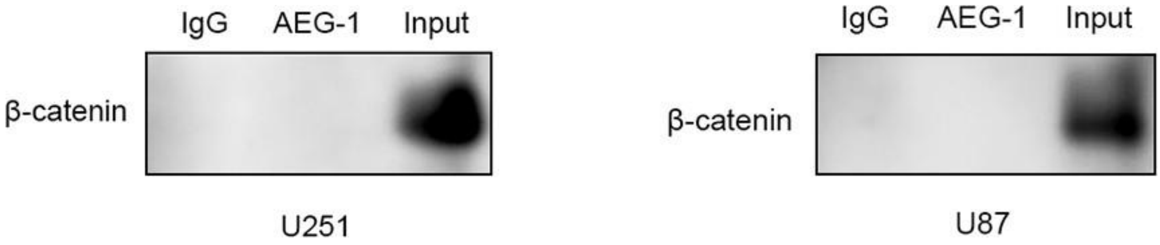

Supplementary Fig. S2

GEPIA **(A)** and Western blot analysis **(B)** showed that *AEG-1* silencing does not affect *MGMT* expression in glioma cells.

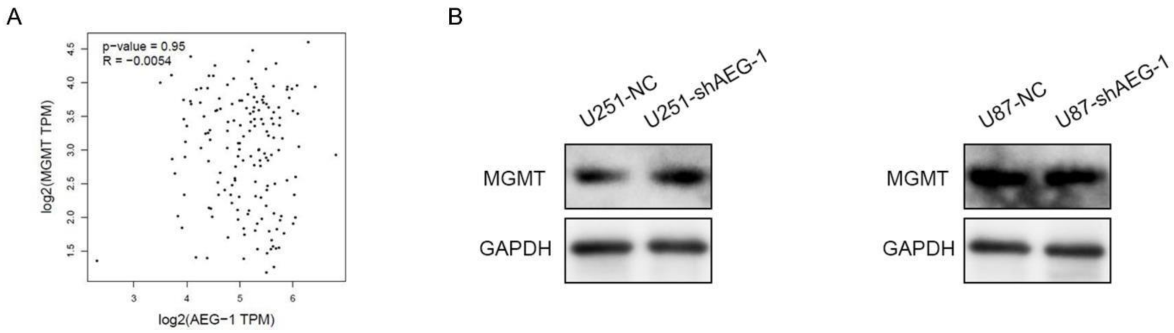

Supplementary Fig. S3

Western blot bands of *AEG-1* and *PD-L1* in NC and sh*AEG-1* glioma cell lines.

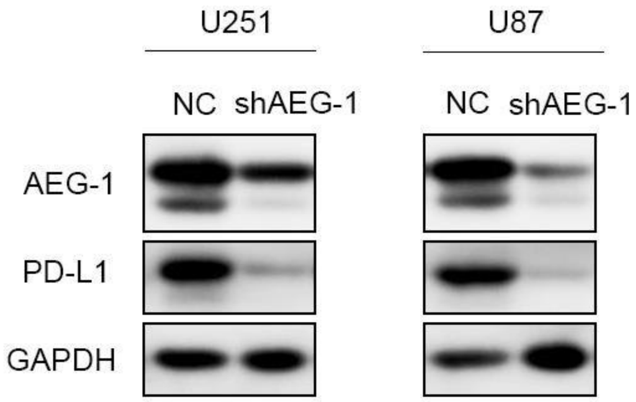

Supplementary Fig. S4

Western blot bands of *CXCL10* in NC and sh*AEG-1* U251 and U87 cell lines. Proteins were separated through standard 15% SDS-PAGE.

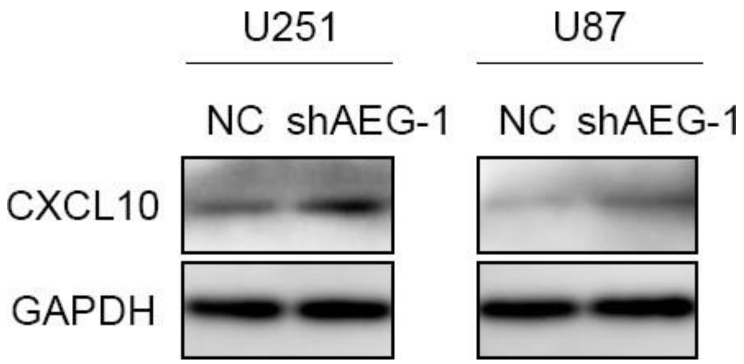

Supplementary Table S1

The top 10 enriched pathways and corresponding differentially expressed genes (DEGs) between negative control and AEG-1 knockdown glioma cells by performing Affymetrix microarray experiments.

**Pathway analysis results**			
Gene Set Name	Gene Number	P-value	Genes
KEGG_PATHWAYS_IN_CANCER	52	7.57E-17	EGFR,RAF1,PIK3R2,ITGA6,FN1,FGFR2,FGFR3,FGFR1,FGF2,FGF5,PRKCA,AKT3,IGF1R,CCND1,PTEN,VEGFA,VEGFC,BIRC3,MYC,TGFB2,TGFBR2,FOS,ABL1,MDM2,CREBBP,LEF1,SMAD4,FZD1,WNT5A,FZD2,FZD4,FOXO1,AR,NKX3-1,RUNX1,BCR,BMP4,IL8,KITLG,CSF2RA,CTNNA2,HIF1A,EPAS1,CEBPA,RARA,RALA,RXRB,SKP2,TRAF1,TRAF3,GLI3,STK36
KEGG_MAPK_SIGNALING_PATHWAY	33	6.53E-08	EGFR,RAF1,FGFR2,FGFR3,FGFR1,FGF2,FGF5,PRKCA,AKT3,MYC,TGFB2,TGFBR2,FOS,RRAS2,MRAS,GNA12,RAP1A,FLNB,RASA1,IL1R1,ZAK,MAP3K5,MAPK

			APK2,BDNF,MEF2C,MAP3K2,MAP4K4,PLA2G12A,CACNA1A,TAB2,DUSP5,STMN1,LAMTOR3
KEGG_REGULATION_OF_ACTIN_CYTOSKELETON	32	2.05E-09	EGFR,RAF1,PIK3R2,ITGA6,FN1,FGFR2,FGFR3,FGFR1,FGF2,FGF5,VAV3,ITGA4,ACTN4,ITGA7,ITGB4,RRAS2,MRAS,GNA12,LIMK1,CFL1,CFL2,ARHGEF12,PIP4K2B,GSN,ARPC1A,SCIN,MYH10,CHRM3,ARHGEF4,EZR,ABI2,ENAH
KEGG_FOCAL_ADHESION	29	2.40E-08	EGFR,RAF1,PIK3R2,ITGA6,FN1,PRKCA,AKT3,IGF1R,CCND1,PTEN,VEGFA,VEGFC,BIRC3,VAV3,ITGA4,ACTN4,ITGA7,ITGB4,RAP1A,FLNB,FYN,SHC2,SHC4,THBS1,THBS2,COL3A1,TNC,ARHGAP5,TLN2
KEGG_ENDOCYTOSIS	25	5.38E-07	EGFR,FGFR2,FGFR3,IGF1R,MDM2,PIP4K2B,ARF6,SMURF2,ADRBK2,ADRBK1,NEDD4,STAM2,ADRB2,AP2B1,RAB11B,AGAP1,PSD3,ACAP2,EHD4,LDLR,VPS36,VTA1,SMAP1,RAB11FIP1,VPS25
KEGG_PURINE_METABOLISM	24	2.08E-07	ENTPD4,POLR1B,POLR2I,POLR3G,POLR3C,POLR3B,POLR1E,PNP,NT5E,NT5C3,PNPT1,CANT1,NME7,ADCY9,GUCY1A2,XDH,PDE1C,ENPP1,PRPS2,ADSSL1,PPAT,ADA,GMPR,PDE4B
KEGG_WNT_SIGNALING_PATHWAY	22	1.02E-06	PRKCA,CCND1,MYC,CREBBP,LEF1,SMAD4,FZD1,WNT5A,FZD2,FZD4,CAMK2D,PLCB4,BTRC,PPP2R5B,PPP2R5E,CSNK1E,FOSL1,DAAM1,CACYBP,SFRP1,TBL1XR1,RUVBL1
KEGG_AXON_GUIDANCE	21	4.25E-07	ABL1,LIMK1,CFL1,CFL2,ARHGEF12,FYN,RASA1,EPHA4,DPYSL2,EFNB3,EPHA2,SRGAP2,PLXNB2,PLXNA1,SEMA6A,ROBO1,SEMA6D,SEMA7A,NRP1,SEMA5A,SEMA3E

KEGG_PYRIMIDINE_METABOLISM	18	5.38E-07	ENTPD4,POLR1B,POLR2I,POLR3G,POLR3C,POLR3B,POLR1E,PNP,NT5E,NT5C3,PNPT1,CANT1,NME7,DPYD,UCK2,UPP1,CMPK2,CTPS2
KEGG_FC_GAMMA_R_MEDIATED_PHAGOCYTOSIS	17	2.26E-06	RAF1,PIK3R2,PRKCA,AKT3,VAV3,LIMK1,CFL1,CFL2,PIP4K2B,GSN,ARPC1A,SCIN,ARF6,RPS6KB2,LYN,PPAP2A,MARCKS

Supplementary Fig. S5 Full-size blots of Fig. 1E.

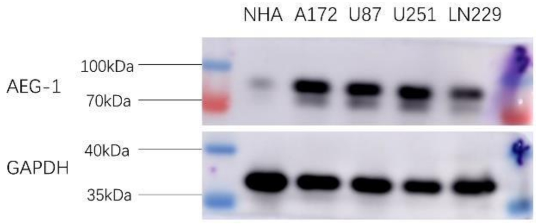

Supplementary Fig. S6 Full-size blots of Fig. 3A and 3B.

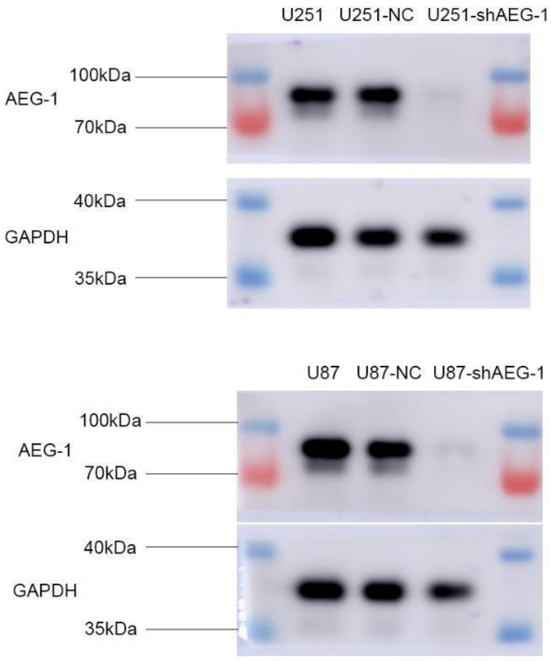

Supplementary Fig. S7 Full-size blots of Fig. 3I.

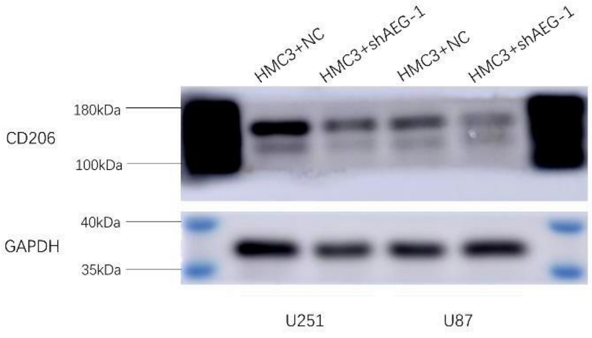

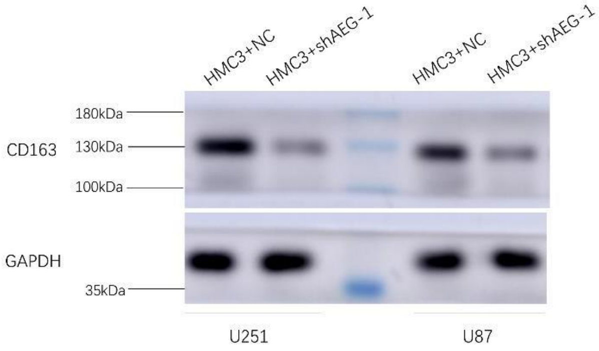

Supplementary Fig. S8 Full-size blots of Fig. 4C and 4D.

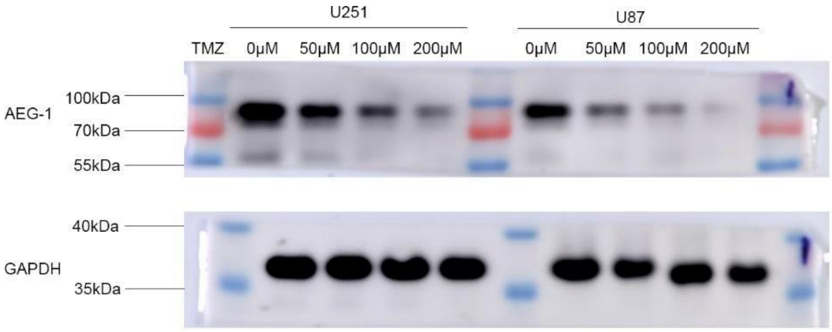

Supplementary Fig. S9 Full-size blots of Fig. 6C.

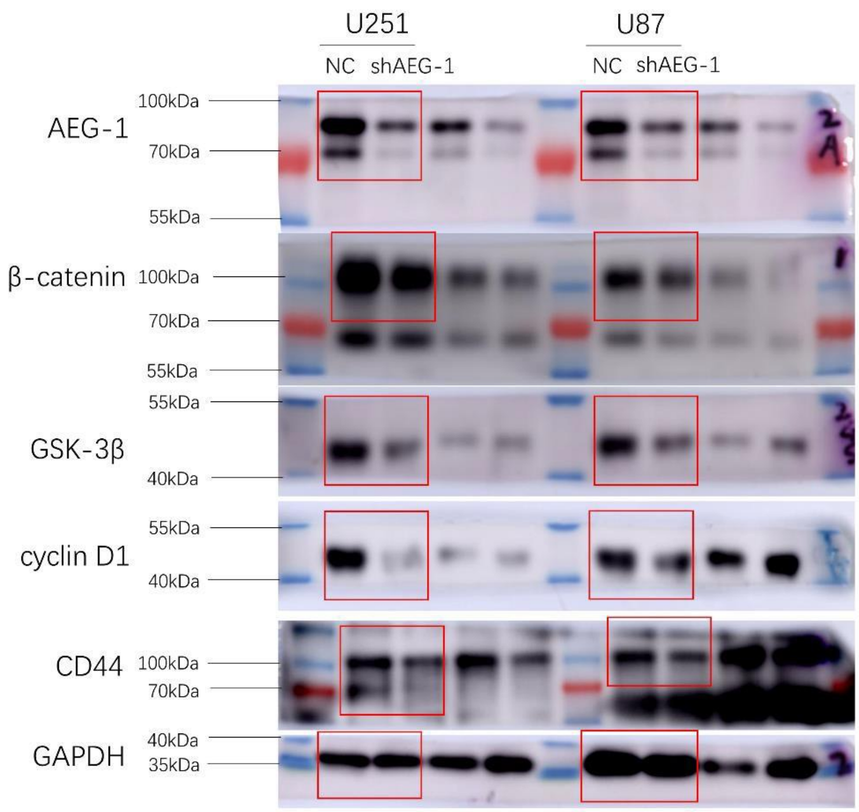

Supplementary Fig. S10   Full-size blots of Fig. 6E.

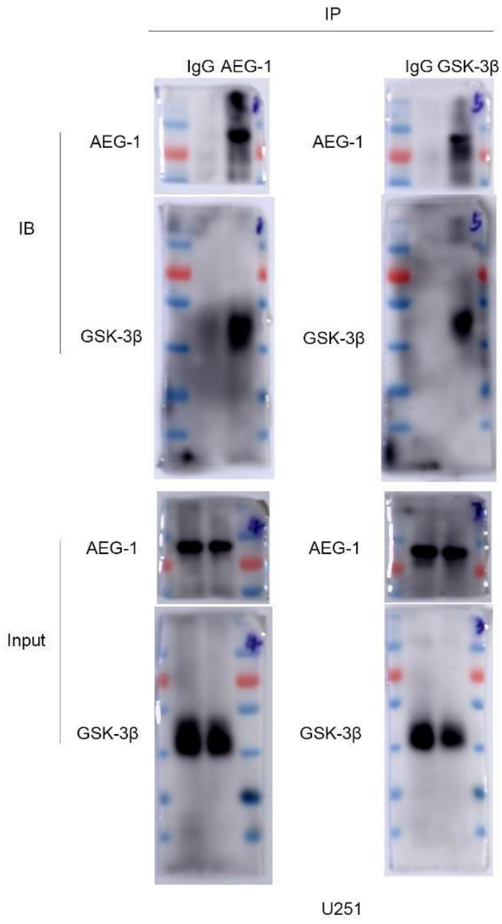

Supplementary Fig. S11 Full-size blots of Fig. 6F.

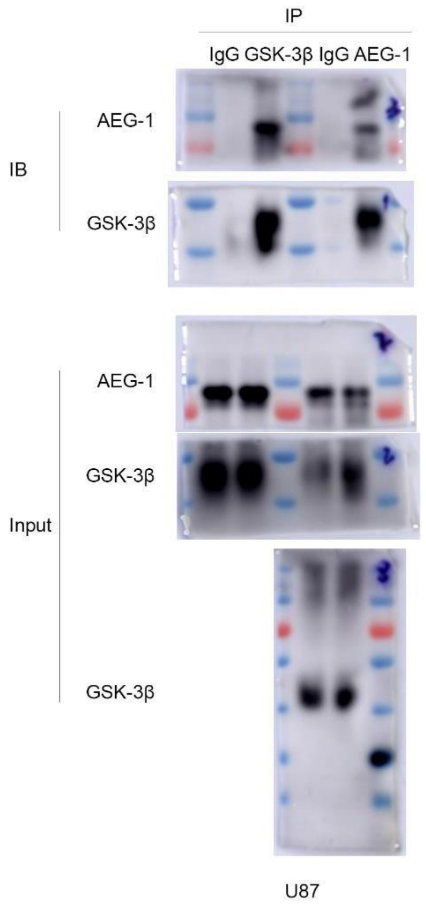

Supplementary Fig. S12 Full-size blots of Supplementary Fig. S1.

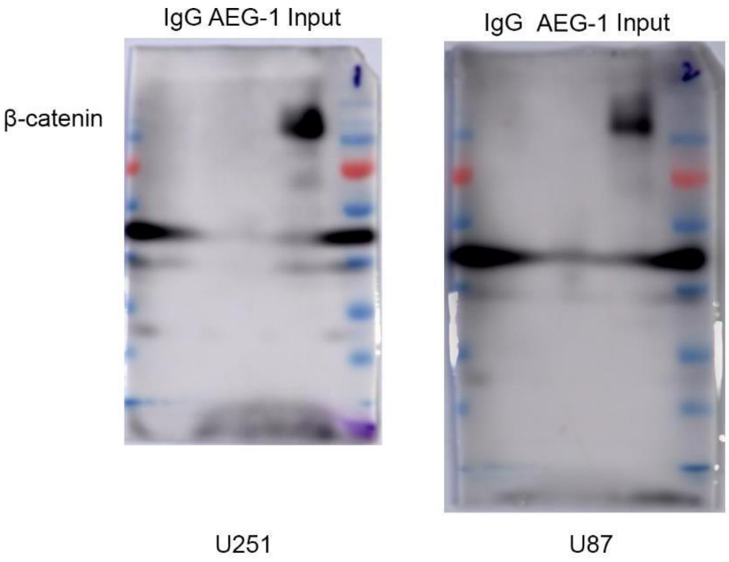

Supplementary Fig. S13 Full-size blots of Supplementary Fig. S3.

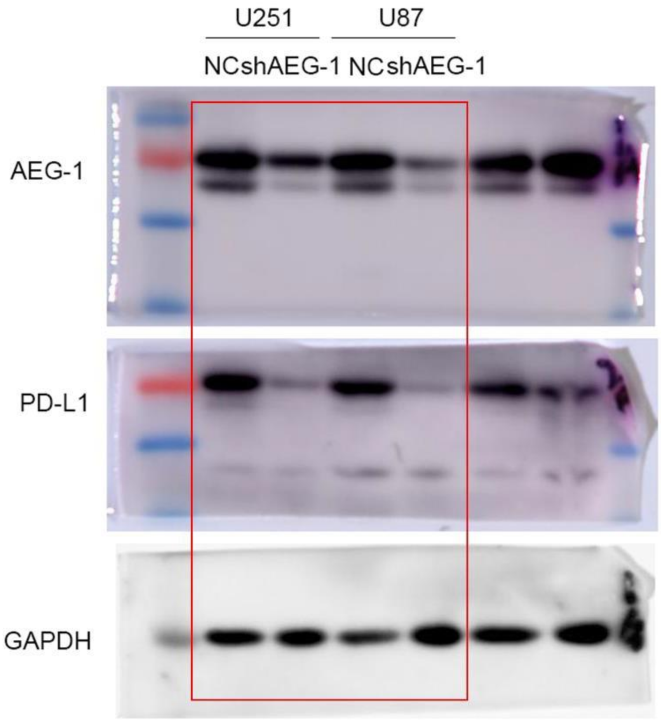

Supplementary Fig. S14 Full-size blots of Supplementary Fig. S4.

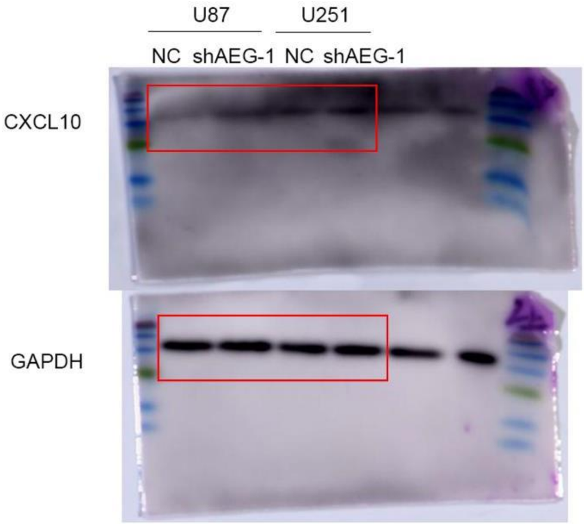

Supplement: Supplementary file 1 — Supplementary Information. [file 41598_2021_96647_MOESM1_ESM.pdf]
